# Association of Body Mass Index with Physical Function and Health-Related Quality of Life in Adults with Arthritis

**DOI:** 10.1155/2013/190868

**Published:** 2013-12-12

**Authors:** Danielle E. Schoffman, Sara Wilcox, Meghan Baruth

**Affiliations:** ^1^Department of Health Promotion, Education, and Behavior, Arnold School of Public Health, University of South Carolina, 800 Sumter Street, Suite 216, Columbia, SC 29208, USA; ^2^Department of Exercise Science, Arnold School of Public Health, University of South Carolina, 921 Assembly Street, Columbia, SC 29208, USA; ^3^Prevention Research Center, Arnold School of Public Health, University of South Carolina, 1st Floor, 921 Assembly Street, Columbia, SC 29208, USA; ^4^Department of Health Science, Saginaw Valley State University, 7400 Bay Road University Center, MI 48710, USA

## Abstract

Arthritis and obesity, both highly prevalent, contribute greatly to the burden of disability in US adults. We examined whether body mass index (BMI) was associated with physical function and health-related quality of life (HRQOL) measures among adults with arthritis and other rheumatic conditions. We assessed objectively measured BMI and physical functioning (six-minute walk, chair stand, seated reach, walking velocity, hand grip) and self-reported HRQOL (depression, stiffness, pain, fatigue, disability, quality of life-mental, and quality of life, physical) were assessed. Self-reported age, gender, race, physical activity, and arthritis medication use (covariates) were also assessed. Unadjusted and adjusted linear regression models examined the association between BMI and objective measures of functioning and self-reported measures of HRQOL. BMI was significantly associated with all functional (*P*s ≤ 0.007) and HRQOL measures (*P*s ≤ 0.03) in the unadjusted models. Associations between BMI and all functional measures (*P*s ≤ 0.001) and most HRQOL measures remained significant in the adjusted models (*P*s ≤ 0.05); depression and quality of life, physical, were not significant. The present analysis of a range of HRQOL and objective measures of physical function demonstrates the debilitating effects of the combination of overweight and arthritis and other rheumatic conditions. Future research should focus on developing effective group and self-management programs for weight loss for people with arthritis and other rheumatic conditions (registered on clinicaltrials.gov: NCT01172327).

## 1. Introduction

Arthritis and other rheumatic conditions are the leading cause of disability in adults in the United States [1]. The negative consequences of arthritis and other rheumatic conditions, including pain, reduced physical ability, depression, and reduced quality of life (QOL) can impact the physical functioning and psychological well-being of those living with the conditions [2–4]. A number of variables have been shown to be associated with arthritis and other rheumatic conditions such as older age, lower physical activity (PA) levels, female gender, and being overweight or obese [5, 6]. Treatment of arthritis and other rheumatic conditions are very costly for insurers and patients alike [7], and given the growing number of people in the United States over the age of 65, arthritis and other rheumatic conditions are set to be an even larger burden on the health care system in the coming years [5]. While about 47.8 million Americans self-reported doctor-diagnosed arthritis and other rheumatic conditions in 2005, this number is expected to reach about 67 million by 2030, meaning that 25% of Americans will have arthritis and other rheumatic conditions [8]. Without effective prevention and treatment strategies, arthritis and other rheumatic conditions will cause significant increases in the already high health care costs weighing on Americans.

High body mass index (BMI) has been shown to be an independent risk factor for arthritis and other rheumatic conditions [6]. Individuals who are overweight or obese are at a greater risk of developing arthritis and higher body weight may also hasten the onset of some forms of arthritis and other rheumatic conditions [6, 9]. A recent study using data from the Behavioral Risk Factor Surveillance Survey (BRFSS) found that the prevalence of arthritis and other rheumatic conditions was highly related to BMI; of those with arthritis, 25.9% were normal weight, 33.7% were overweight, and 43.7% were obese [6]. Unfortunately, rates of obesity continue to rise, with recent data showing that 33.3% of American adults are overweight, and an additional 35.9% are obese [10].

Past research has demonstrated a relationship between arthritis and other rheumatic conditions and numerous physical and psychosocial impairments, such as difficulty with activities of daily living, decreased PA, impaired QOL, and loss of quality-adjusted life years [2, 3, 11]. Additionally, obesity has been shown to be associated with decreased PA, decreased health-related quality of life (HRQOL), and an increased risk of depression [12–14]. However, a very small number of studies have specifically looked at the relationship between BMI and the symptoms of arthritis and other rheumatic conditions in adults [3]. BMI was shown to be associated with increased symptom severity and decreased QOL in a sample of participants with fibromyalgia and decreased physical functioning in individuals with osteoarthritis [15, 16]. No studies to date have examined the association between BMI and objectively measured, laboratory-tested physical function measures (e.g., six-minute walk and chair stand). Furthermore, studies have not examined the relationship between BMI and various QOL measures in diverse samples of adults with different types of arthritis and other rheumatic conditions.

While the medical treatment for arthritis and other rheumatic conditions varies widely by subtype, the public health interventions for this disease utilize strategies that are applicable regardless of arthritis type. The Centers for Disease Control and Prevention (CDC) has validated a case definition of arthritis for public health interventions that has been used in BRFSS and the National Health Interview Study (NHANES) since 1992 [17]. This definition includes all community-dwelling adults with self-reported doctor-diagnosed arthritis, including all types of arthritis and rheumatic conditions [17]. In a recent article, arthritis experts from the CDC urged researchers and practitioners to work together to develop public health strategies to reduce the burden of arthritis (as broadly defined by the case definition), through strategies programs such as self-management, weight loss, and PA promotion [17].

The purpose of this investigation is to describe the relationship between BMI, objectively measured physical function, and QOL-related measures in a racially diverse sample of adults, representing a broad range of ages and arthritis types. Using a large sample and a variety of performance-based and self-report measures, we hypothesize that individuals with a higher BMI will demonstrate poorer performance on measures of physical function and report greater impairments on self-reported QOL measures.

## 2. Methods

Data in this study are cross-sectional and were taken from the baseline measurement visit (prior to randomization) of participants enrolled in a randomized trial of two self-directed programs for arthritis management. A priori power calculations indicated that 300 participants were necessary to detect small group differences (effect sizes *d* = 0.23–0.38) with 80% power for the primary outcomes (e.g., pain, fatigue, stiffness, and gait speed). To account for attrition in the clinical trial, the recruitment goal was set at 400 participants. A number of strategies were used to recruit participants into the study, with the most successful being worksite listservs and newspaper advertisements. Interested participants contacted the study office and completed a phone screen to assess eligibility status.

Participants were adult community members with self-reported, doctor-diagnosed arthritis, and other rheumatic conditions. Participants were eligible to take part in the study if (1) they answered “yes” to the question: “Have you EVER been told by a doctor or other health care professional that you have some form of arthritis, rheumatoid arthritis, gout, lupus, or fibromyalgia?” (this question uses the CDC definition of arthritis and is used in the BRFSS) [17]; (2) they reported at least one symptom of arthritis (joint pain, stiffness, tenderness, decreased range of motion, redness and warmth, deformity, crackling or grating, and fatigue); (3) they were 18 years of age or older; (4) they are not diabetic and taking insulin; (5) they did not have uncontrolled hypertension; (6) they were able to participate in PA (as measured by the Physical Activity Readiness Questionnaire (PAR-Q)) [18]; (7) they were sufficiently inactive at the time of enrollment (defined as engaging in <3 days per week of at least 30 minutes of aerobic activity and <2 days per week of at least 20 minutes of strength training).

Participants were ineligible if (1) they had a fall in the past year that required medical assistance; (2) they were pregnant, breastfeeding, or planning to become pregnant in the next year (women); (3) they were a diabetic and taking insulin; (4) they could not walk longer than 3 minutes without taking a rest; (5) they could not stand without assistance for more than 2 minutes; (6) they could not sit in chair without arms for more than 5 minutes; (7) they were already physically active (aerobic activities ≥3 days/week for ≥30 minutes/day or strength training ≥3 days/week for ≥20 minutes/day). The Physical Activity Readiness Questionnaire (PAR-Q) [18] was also administered and participants endorsing any items, with one exception, were excluded. Participants were not excluded if they took medication for hypertension; however, they were excluded if they had uncontrolled hypertension (≥160/100).

Of the initial phone inquiries (*n* = 1112), most participants completed a phone screen (*n* = 923), and about half were eligible and interested in the project (*n* = 10 participants were eligible but no longer interested) and were scheduled for a baseline visit (*n* = 545); 401 of these participants completed a baseline visit and were randomized to a self-management program, whereas 135 did not attend their baseline visit, and 9 were excluded at the baseline visit prior to randomization (7 for medical contraindications, 2 based on staff discretion). The 368 participants deemed ineligible after the phone screen were ineligible for a variety of reasons (e.g., regular exerciser and medical condition). Full details about recruitment for the randomized trial and the flow of participants have been reported elsewhere [19].

Prior to the scheduled measurement session, participants were mailed a survey that assessed sociodemographic characteristics; PA, dietary, and other health-related practices; QOL; and arthritis-related characteristics. Participants brought the completed survey to the session. Participants completed an informed consent form that was approved by the Institutional Review Board at the University of South Carolina. Upon providing consent to participate, staff administered physical measurements including height, weight, blood pressure, and physical function tests; participants received a $20 cash incentive from completion of the session. This study is registered on clinicaltrials.gov, trial identifier: NCT01172327.

### 2.1. Measures

#### 2.1.1. Sociodemographic/Health-Related

Participants reported their age, gender, race, the highest grade or years of education completed. Objectively measured height and weight were obtained by trained staff. Body mass index was calculated as kg/m^2^ using standard procedures and cut points [20].

#### 2.1.2. Functional Exercise Capacity

The six-minute walk test was used to measure functional exercise capacity. A 38 meter walking course was marked with cones in a level, carpeted hallway. Participants were instructed to walk as quickly as possible (not run) for 6 minutes. They were allowed to use their usual assistive devices during the test and were allowed to slow down, stop, or rest as necessary. A staff member called out the time every minute (e.g., you have 3 minutes to go) and encouraged participants in a standardized manner using one of two phrases, “you are doing well” or “keep up the good work.” The score was the total distance walked (in meters) in 6 minutes. This test has been shown to be valid and reliable [21, 22].

#### 2.1.3. Lower Body Strength

Lower body strength was measured using the 30-second chair stand. Participants were directed to sit in the middle of a standard chair with their back straight, feet flat on the floor, with their hands on the opposite shoulder crossed at the wrist. On the signal, participants rose to a full stand and returned to a fully seated position, without using their arms. One practice of 1–3 repetitions was followed by one 30-second trial [23]. The score was the total number of unassisted stands during the 30-second time frame. This measure has been shown to be valid and have good test-retest reliability in a sample of older adults [24].

#### 2.1.4. Lower Body Flexibility

Lower body flexibility was measured using the seated reach test. Participants removed their shoes and sat on a raised mat with their legs extended, knees straight, and feet positioned against a sit and reach box. With their arms outstretched, hands overlapping, and middle fingers even, participants slowly bent forward, reaching as far forward as possible toward their toes and pushing a marker forward. Assistive blocks were used (10, 20, and 30 cm) if participants could not reach the zero position. Participants were given 2 practice and 3 test trials. The score was the total distance reached minus the assistive block (if used) to the nearest 0.5 cm, using the best of the three trials. Higher position scores are more favorable. This measure has shown acceptable validity (for hamstring flexibility) in a sample of middle-aged to older adults [25].

#### 2.1.5. Gait

The GAITRite (CIR Systems, Havertown, PA), a portable walking mat with software, was used to measure kinematic parameters of the gait cycle. Participants walked on the instrumented walkway without shoes at their normal walking pace. Sufficient distance was provided at the start and end of the walkway to insure a normal walking velocity. Participants completed three test trials, and the three trials were averaged. Participants needing an assistive device were allowed to use it during data collection. The primary measure of interest was gait speed, which was measured in centimeters/second but converted to meters/second. The GAITRite has been previously validated with a three-dimensional motion analysis system. A variety of gait parameters were evaluated and showed an excellent level of agreement indicating the GAITRite system is a valid technique for quantifying both averaged and individual parameters of gait [26]. Test-retest reliability was found to be high across a number of reported variables [27].

#### 2.1.6. Upper Body Strength

Grip strength was measured on the dominant hand using a Jamarhand dynamometer, positioned in the no. 2 ring, (Lafayette Instruments, Lafayette, IN) and was measured in kilograms. Participants stood with their dominant arm at their side (not touching the body), elbow bent to 90 degrees, wrist in the neutral position, and thumb superior. On the signal, participants squeezed the dynamometer with as much force as possible. No motivational coaching was provided during the trials. Participants were given one practice and three tests (with a 10–20 second rest in between each) trials. The best of three trials was used as the score. This measure has been shown to be reliable [28] and valid [29].

#### 2.1.7. Depressive Symptoms

The 10-item Center for Epidemiological Studies Depression Scale (CES-D) [30–32] was used to measure symptoms of depression. On a scale of 0 (rarely or none of the time) to 3 (most or all of the time), participants rated the frequency with which they experienced 10 symptoms of depression during the past week. Responses were summed to yield a score ranging from 0 to 30, with a higher score indicating greater depressive symptoms. This measure has been shown to be reliable and valid [31, 33, 34].

#### 2.1.8. Symptoms of Arthritis: Pain, Fatigue, Stiffness

Using a Visual Numeric Scale [35], participants rated their arthritis symptoms in the past 2 weeks on a numeric scale from 0 (no symptoms) to 10 (severe symptoms). Separate items were used to evaluate generalized pain, stiffness, and fatigue. This measure has been shown to be sensitive to detecting reduction in pain after the completion of an arthritis self-management course [36].

#### 2.1.9. Disability

The 20-item Health Assessment Questionnaire (HAQ) Disability Index was used to measure disability in eight categories of daily activities (i.e., dressing, arising, eating, walking, hygiene, reach, grip, and common activities). On a scale of 0 (without any difficulty) to 3 (unable to do), participants reported the amount of difficulty they had in performing two or three specific activities (in each category) over the past week. Each of the eight categories was assigned a score based on the highest score of any activity within the category. If the category score was lower than a 2 but a participant reported usually using a device or aid to perform the activity, the score was increased to a 2. The total score was the mean of the eight categories. Scores ranged from 0 to 3, with a higher score indicating higher impairment. This measure has been shown to be valid [37] and reliable [38].

#### 2.1.10. Quality of Life

The Centers for Disease Control and Prevention's 4-item Healthy Days Core Module measured HRQOL [39]. Participants reported the number of days (in the past 30) that their physical health was not good, their mental health was not good, and the number of days that poor physical or mental health kept them from doing usual activities. The reliability and validity of this measure have been previously established [39, 40].

#### 2.1.11. Self-Reported PA

The Community Health Activities Model Program for Seniors (CHAMPS) questionnaire, originally developed for older adults, is a 42-item self-report measure of PA [41]. It includes activities typically undertaken for exercise, activities undertaken in the course of one's day that are physical in nature and recreational activities that provide PA. For each item, participants reported whether or not they had engaged in the activity in a typical week in the past 4 weeks, the number of times per week, and the total number of hours per week (in 6 categories ranging from “less than 1 hour a week” to “9 or more hours per week”). This measure has been shown to be valid [42], have acceptable test-retest reliability [42], and be sensitive to change [41]. Total hours per week of MVPA (≥3.0 METs) per week were calculated. Calculations were based on the MET values reported in the Ainsworth et al. [43] Compendium, adjusted for the recommendations made by Stewart et al. [41].

#### 2.1.12. Medication

Participants were asked to report if they were currently taking Tylenol or acetaminophen, nonsteroidal anti-inflammatory drugs (NSAIDS), COX-2 inhibitors, oral steroids, narcotic pain relievers, or any other over-the-counter and prescription medications for their arthritis (open-ended question). Medications listed in the open-ended questions were coded and reclassified to the above mentioned categories if applicable. Given the common reporting of the use of disease-modifying antirheumatic drugs (DMARDS) (in the open-ended question), an additional category of drugs was created. If participants reported current use or at least one day of use of any one or more of these six categories of drugs in the past 7 days, they were considered to be using arthritis medication.

### 2.2. Analysis

Basic descriptive statistics included frequencies and means of key survey, selected demographic, and health-related variables. Linear regression models examined the relationship between BMI, physical functioning, and arthritis symptoms. A separate model was conducted for each physical function measure (six-minute walk test, 30-second chair stand, seated reach, velocity, and grip strength) and each HRQOL variable (depression, pain, fatigue, stiffness, HAQ total, QOL, physical, and QOL, mental). Unadjusted models were first run for all functional and HRQOL variables. Next, adjusted models were run for the same dependent variables, controlling age, gender, race (white, non-white), MVPA, and arthritis medication use. PROC GLM was used to run all regression models (SAS version 9.2; SAS Institute, Cary, NC, USA). PROC GENMOD was used to run negative binomial models for variables with nonnormal distributions. Results of the negative binomial models were similar to those of the linear regression models (in terms of direction and significance of the results); thus, for the sake of simplicity and ease of interpretation, linear regression results are reported here. Analyses used a 0.05 level of statistical significance.

## 3. Results

Table 1 presents demographic, weight status, physical function, and HRQOL variables for all participants (*N* = 401). Participants ranged in age from 19 to 87 years, with a mean age of 56.3 ± 10.7 years. The sample was predominantly female (85.8%), and a majority had a college degree (60.8%). The average BMI was 33.1 ± 8.3 kg/m^2^ and 56.9% were obese (BMI ≥ 30 kg/m^2^) [43].  Table 2 presents the overall model *F* test, partial *F* test for BMI, and model *R* ^2^ for each unadjusted and adjusted regression model measuring the association between BMI and each of the functional measures; Table 3 presents the models for the association between BMI and each of the HRQOL measures. BMI was significantly associated with all of the functional measures (*P*s ≤ 0.007) and all of the HRQOL measures (*P*s ≤ 0.03) in the unadjusted models. A higher BMI was associated with more impaired scores for all functional and QOL measures, with the exception of grip strength, where a higher BMI was associated with greater grip strength. Associations between BMI and all functional measures remained significant in the adjusted models (*P*s ≤ 0.001). Associations between BMI and most of the QOL measures also remained significant in the adjusted models (*P*s ≤ 0.05); associations between BMI and depression (*P* = 0.055) and QOL, physical (*P* = 0.15), were no longer significant but approached significance in the predicted direction.

## 4. Discussion

The impaired functioning caused by arthritis and other rheumatic conditions can be debilitating, both physically and mentally. Our findings suggest that being at an unhealthy weight may further exasperate these impairments. BMI was associated with greater impairments in a number of physical function and QOL related measures, even after controlling age, gender, race, MVPA, and arthritis medication use, speaking to the robustness of these relationships.

Overall, BMI was highly associated with impaired physical function as measured by a variety of objective tests of function. These functional tests measure physical ability across a wide variety of domains, designed to measure the capacity for independent functioning of the individual [23]. A higher BMI was associated with shorter distances on the six-minute walk test, fewer chair stands, shorter seated reach, and lower walking speed, demonstrating participants' impaired functional exercise capacity, lower body strength, flexibility, and walking speed as compared to participants with a lower BMI. The physical disability already experienced by many suffering from arthritis and other rheumatic conditions appears to be magnified by the additional burden of a higher BMI. Higher levels of arthritis disability have been shown to be linked to increased medical costs, hospital visits, comorbid conditions, and other serious medical consequences [7, 8]. Together, these findings support the need for efforts aimed at decreasing BMI among individuals with arthritis and other rheumatic conditions.

A higher BMI was also associated with some measures of HRQOL, including strong associations with higher self-reported pain, fatigue, stiffness, disability, and QOL, mental. Numerous studies have shown an association between arthritis and lower HRQOL and disability [1, 44] in a smaller range of measures, but the results of this study demonstrate the widespread mental and physical impacts of BMI. In this study, greater physical and mental impairments were found among arthritic individuals with a higher BMI, across a range of both objectively measured physical function and self-reported measures of HRQOL.

Grip strength was the only functional measure to be *positively *associated with BMI, a finding consistent with other recent researches [45]. A recent study of grip strength in the general population found that while a higher BMI was related to greater grip strength in adults aged 30 to 70 years, there was an inverse relationship among those over 70 years of age, where a higher BMI was associated with lower grip strength [46]. Associations between BMI and depression and QOL, physical, were not statistically significant in the adjusted models but approached significance in the predicted direction. The relationship between BMI and QOL observed in this study was not as strong as what has been observed in other recent studies; for example, one study found that BMI was independently related to impaired QOL in adults with rheumatoid arthritis [3].

It is important to note that the definition of functional measures used in the current study only represents a portion of what the World Health Organization (WHO) has defined as physical function and the causes of disability [47]. The WHO published a framework for understanding the determinants of disability, which includes body functions and structure, and activity limitations and participation restrictions [47]. All of these elements interact within the context of the environment that the individual lives in, and it is within this framework that the experience of disability can be better understood [47]. The measures used in the present study were all obtained at the level of body functions, thus only capturing a part of the disability picture. It is possible that the full impact of BMI-related disability for people with arthritis and rheumatic conditions is more complex than the measures we used. A recent study from the related field of joint disease looked at posttotal hip replacement surgery patients, examining the influence of overweight/obesity on physical function and HRQOL but also looking at more complex effects of complications and comorbidities [48]. The results showed that while the influence of overweight/obesity on physical function and HRQOL was minimal, there was a substantial negative effect of postsurgical complications and comorbidities [48]. This indicates the need to include more variables to describe the complex relationships between physical function and disability at multiple levels of influence, including comorbidities, in order to best model its impact on physical and emotional well-being.

Given that the prevalence of obesity and arthritis and other rheumatic conditions continue to rise in the United States, it is important that researchers and clinicians work to find strategies to improve the QOL and level of functioning of affected individuals. Despite the high correlation between being overweight and having arthritis and other rheumatic conditions, few interventions have investigated the links between the two conditions or offered suggestions for future treatment programs. Some research has shown the benefit of weight loss for the improvement of obesity-related conditions, including the alleviation some of the symptoms of arthritis [49]. Unfortunately, few overweight individuals receive diet, exercise, or weight loss counseling from their primary care physician [50]. One study targeting obese individuals with arthritis found that only 43% received weight loss counseling from their physician [9]. However, this study also found that when individuals did receive weight loss advice from their physician, they were more likely to lose weight than those patients that did not receive the advice [9]. Another study found that physician advice was independently associated with patient engagement in arthritis self-management strategies of any kind [51]. These studies demonstrate the influence physicians may have in arthritic populations, perhaps making them an important partner in interventions aimed at improving health behaviors in this population.

In addition to weight loss, getting sufficient PA has been recommended as an arthritis self-management strategy [51]. Increased PA could lessen the symptoms of arthritis and other rheumatic conditions and promote weight loss. However, often times, the pain, stiffness, and other symptoms that accompany arthritis and other rheumatic conditions deter people from being active [52]. On average, individuals with arthritis are insufficiently active and are even less likely to be active than people without arthritis [53]. It is possible that the excess weight prevents some arthritic individuals from being active; therefore, losing weight may actually lead to increases in PA. Unfortunately, persons with arthritis have expressed that there is a lack of exercise programs available that are suitable to their specific needs [52]. One aim of the current trial is to examine the effectiveness, safety, and participant satisfaction with a self-directed PA program designed for adults with arthritis. Results from this trial will provide insight into what aspects of a program may be the most acceptable in this population.

There is a paucity of research examining factors associated with successful weight loss and increased PA in adults with arthritis and other rheumatic conditions. Additional studies examining these predictors would help researchers and clinicians develop more effective programs that meet the needs of this at risk population. From a public health perspective, effective group-based programs or low-cost self-management programs are particularly appealing, as a large number of people with arthritis and other rheumatic conditions could be helped with relatively little resources.

Our findings should be interpreted in the context of some recognized limitations. First, because this is a cross-sectional study, we are unable to draw causal inferences and can only suggest relationships between BMI and physical function and HRQOL. Second, our study had an underrepresentation of men, although our sample was similar to other recent arthritis studies [3, 15]. National surveys show that women have higher rates of arthritis than men, so the gender representation in our sample is not surprising [8]. Finally, our sample was limited to insufficiently active people, and it is possible that the associations found between BMI and physical function and HRQOL are not generalizable to physically active people with arthritis and other rheumatic conditions. However, even within our sample, there was a great deal of variability in terms of PA participation. Despite these limitations, the relationship observed between BMI and physical function and HRQOL in this study offers evidence of the many areas of life that might be affected by the combination of being overweight and having arthritis and other rheumatic conditions. Strengths of the study sample include the relatively large number of participants (*N* = 401), the age range of participants (19 to 87 years), the use of objective measures of physical functioning and BMI, and the variety of physical function HRQOL measures collected.

In conclusion, BMI was strongly associated with physical function and HRQOL measures in a sample of adults with arthritis and other rheumatic conditions. With the rising rates of obesity and arthritis and other rheumatic conditions, management strategies for both chronic conditions are imperative. Physicians can aid in this effort by offering more frequent support and advice for weight loss to their overweight patients with arthritis to help avoid the disabling combination of these conditions. Future research is needed to develop effective group and self-management programs for weight-loss in people with arthritis and other rheumatic conditions.

## Figures and Tables

**Table 1:** Baseline demographics, arthritis medication usage, weight status, physical function, and health-related quality of life measures (*N* = 401)^a^.

	*n*	% or mean (SD)^b^	Range
*Demographic characteristics *			
Age, years	401	56.3 (10.7)	19–87
Gender	401		
Men	57	14.2	
Women	344	85.8	
Race	400		
White	256	64.0	
African American	141	35.3	
American Indian	2	0.5	
Multiracial	1	0.3	
Education	400		
Less than a high school graduate	6	1.5	
High school graduate or GED	46	11.5	
Some colleges	105	26.3	
College graduate	243	60.8	
Employment status	399		
Employed for wages	258	64.7	
Self-employed	15	3.8	
Out of work	14	3.5	
A homemaker	7	1.8	
A student	3	0.8	
Retired	90	22.6	
Unable to work	12	3.0	

*Arthritis medication usage *	401		
≥1 arthritis medication	341	85.0	
Acetaminophen	139	34.7	
NSAIDS	254	63.3	
Steroids	32	8.0	
Narcotics	67	16.7	
DMARD	46	11.5	

*Weight status *			
BMI, kg/m^2^	401	33.1 (8.3)	15.8–60.7
Underweight (BMI < 18.5)	1	0.25	
Normal weight (18.5 ≤ BMI < 25)	58	14.5	
Overweight (25 ≤ BMI < 30)	114	28.4	
Obese (BMI ≥ 30)	228	56.9	

*Physical activity *			
Hrs/wk of moderate to vigorous physical activity	401	3.4 (3.8)	0–25.5
Physical function measures			
Six minute walk, m	399	494.1 (91.2)	151.5–721.6
Chair stands, no. stands	401	10.0 (3.5)	0–24.0
Seated reach, cm	399	21.7 (9.9)	−11.5–49.0
Walking velocity, m/s	397	1.1 (2.2)	0.4–1.7
Grip strength, kg	401	27.1 (10.2)	4.5–74.0

*Health-related quality of life *			
Depression^c^	401	6.5 (5.1)	0–28.0
Stiffness^d^	400	5.3 (2.5)	0–10.0
Pain^d^	401	4.7 (2.3)	0–10.0
Fatigue^d^	401	5.0 (2.6)	0–10.0
Disability^e^	401	0.6 (0.5)	0–2.0
Quality of life, physical^f^	400	6.9 (9.1)	0–30.0
0 days	107	26.75	
1 day–14 days	222	55.5	
>14 days	71	17.75	
Quality of life, mental^f^	399	5.2 (7.9)	0–30.0
0 days	148	37.09	
1 day–14 days	196	49.13	
>14 days	55	13.78	

^a^Some *N*s are less than 401 due to participant refusal to complete measure.

^b^May not add to 100% due to rounding.

^c^Scores range from 0 to 30, with higher score indicating greater depressive symptom.

^d^Scores range from 0 to 10, with higher scores indicating more severe symptoms.

^e^Scores range from 0 to 3, with high scores indicating higher impairment.

^f^Scores range from 0 to 30, with higher score indicating more bad days.

**Table 2:** Unadjusted and adjusted associations between body mass index and physical function measures.

	Model 1^a^			Model 2^b^		
	BMI coeff (*P*)	Model *F* (*P*)	Model *R* ^2^	BMI coeff (*P*)	Model *F* (*P*)	Model *R* ^2^

Physical function measures						
Six-minute walk	−5.16 (<0.0001)	109.96 (<0.0001)	0.22	−5.10 (<0.0001)	35.78 (<0.0001)	0.35
Chair stands	−0.10 (<0.0001)	23.70 (<0.0001)	0.06	−0.08 (0.0001)	13.10 (<0.0001)	0.17
Seated reach	−0.36 (<0.0001)	39.22 (<0.0001)	0.09	−0.35 (<0.0001)	12.58 (<0.0001)	0.16
Walking velocity	−0.009 (<0.0001)	59.99 (<0.0001)	0.13	−0.009 (<0.0001)	17.40 (<0.0001)	0.21
Grip strength	0.17 (0.007)	7.43 (0.007)	0.02	0.16 (0.001)	45.20 (<0.0001)	0.41

^a^Model 1: unadjusted; it contains only BMI (model df = 1).

^b^Model 2: adjusted for age, gender, race, moderate to vigorous intensity physical activity, and arthritis drug usage (model df = 9).

**Table 3:** Unadjusted and adjusted associations between body mass index and health-related quality of life measures.

	Model 1^a^			Model 2^b^		
	BMI coeff (*P*)	Model *F* (*P*)	Model *R* ^2^	BMI coeff (*P*)	Model *F* (*P*)	Model *R* ^2^

Health-related quality of life						
Depression	0.07 (0.02)	5.16 (0.02)	0.01	0.06 (0.055)	4.19 (0.0004)	0.06
Stiffness	0.07 (<0.0001)	23.81 (<0.0001)	0.06	0.06 (<0.0001)	7.70 (<0.0001)	0.11
Pain	0.05 (0.0003)	13.42 (0.0003)	0.03	0.04 (0.007)	5.26 (<0.0001)	0.07
Fatigue	0.06 (<0.0001)	16.76 (<0.0001)	0.04	0.06 (0.0006)	7.94 (<0.0001)	0.11
Disability (HAQ total)	0.01 (0.0004)	12.62 (0.0004)	0.03	0.01 (0.001)	6.05 (<0.0001)	0.08
Quality of life, physical	0.12 (0.03)	4.62 (0.03)	0.01	0.08 (0.15)	3.58 (0.002)	0.05
Quality of life, mental	0.12 (0.01)	6.00 (0.01)	0.01	0.1 (0.05)	3.97 (0.0007)	0.06

^a^Model 1: unadjusted; it contains only BMI (model df = 1).

^b^Model 2: adjusted for age, gender, race, moderate to vigorous intensity physical activity, and arthritis drug usage (model df = 9).
